# Are female students in general and nursing students more ready for teamwork and interprofessional collaboration in healthcare?

**DOI:** 10.1186/1472-6920-11-15

**Published:** 2011-04-21

**Authors:** Margaretha Wilhelmsson, Sari Ponzer, Lars-Ove Dahlgren, Toomas Timpka, Tomas Faresjö

**Affiliations:** 1 Department of Medical and Health Sciences/Community Medicine, Faculty of Health Sciences, Linköping University, SE-581 83 Linköping, Sweden; 2 Department of Clinical Science and Education, Södersjukhuset, Karolinska Institutet, Stockholm, Sweden; 3 Department of Behavioural Sciences and Learning, Faculty of Arts and Science, Linköping University, SE-581 83 Linköping, Sweden

## Abstract

**Background:**

Interprofessional Education (IPE) is now spreading worldwide and many universities are now including IPE in their curricula. The aim of this study was to investigate whether or not such student characteristics as gender, previous working experience in healthcare, educational progress and features of the learning environment, such as educational programmes and curriculum design, have an impact on their open-mindedness about co-operation with other professions.

**Methods:**

Medical and nursing students at two Swedish universities were invited to fill in the Readiness for Interprofessional Learning Scale (RIPLS). Totally, 955 students were invited and 70.2% (n = 670) participated in the study. A factor analysis of the RIPLS revealed four item groupings (factors) for our empirical data, but only one had sufficient internal consistency. This factor was labelled "Team Player".

**Results:**

Regardless of the educational programme, female students were more positive to teamwork than male students. Nursing students in general displayed more positive beliefs about teamwork and collaboration than medical students. Exposure to different interprofessional curricula and previous exposure to interprofessional education were only to a minor extent associated with a positive attitude towards teamwork. Educational progress did not seem to influence these beliefs.

**Conclusions:**

The establishment of interprofessional teamwork is a major challenge for modern healthcare. This study indicates some directions for more successful interprofessional education. Efforts should be directed at informing particularly male medical students about the need for teamwork in modern healthcare systems. The results also imply that study of other factors, such as the student's personality, is needed for fully understanding readiness for teamwork and interprofessional collaboration in healthcare. We also believe that the RIPL Scale still can be further adjusted.

## Background

At the beginning of their education, students in health and social care often have a strong identification with the professional group into which they are going to graduate, and many of them also tend to be open-minded about collaboration with other professions [1]. A recent study reported that students who had been exposed to Interprofessional Education (IPE) curricula were more confident at qualification about their communicative skills, their interprofessional relationships and their professional interactions [2]. The same study also reported a positive connection between students' perceptions of their relevant skills and their interprofessional relationships compared to students who only had uniprofessional education [2]. These results support the perception of the advantage of an early introduction to IPE for students [2]. In a Swedish study, however, no differences were found in attitudes to collaboration between doctors and nurses among medical students (both first-year and final-year students) who had been exposed to IPE curricula and those who had received a more traditional education [3].

Even so, openness to interprofessional collaboration in healthcare has also been reported to be contingent on other circumstances than the educational environment. Historically, the nurse-doctor relationship is complicated in a traditional hierarchical healthcare system comprising professional groups who often have stereotypical perceptions of each other [4]. The social identity theory explains inter-group discrimination and describes an inter-personal and inter-group continuum. Stereotyping, which can hinder effective interprofessional collaboration between professions, may already be apparent at the undergraduate level and constitutes a barrier to effective interprofessional education [5].

Female nurses have been reported to be more willing to serve and support male doctors than female doctors [6]. Female nurses also tend to approach female doctors on a more egalitarian basis, while being more hostile towards them. These findings suggest an imbalance based on both gender and traditional hierarchical structures in healthcare systems [6]. Female medical students, both those who have been exposed to IPE curricula and those who have not, have been reported to be generally more positive to collaboration between nurses and doctors [3]. In teamwork, however, women perform best when competing in pure female teams, whereas men perform best when women are present in a competitive environment [7]. No differences regarding willingness to participate in teamwork have been demonstrated between students in the early and late stages of education [2,3]. Earlier experiences of higher education and age (older students) had a negative influence on attitudes to IPE [2]; while earlier experiences of working in healthcare had no impact on attitudes towards collaboration between nurses and doctors [3].

The countries that are known to be the most advanced in training students in IPE are the United Kingdom, Canada, Australia, the Nordic countries and Japan. However, only a few universities in these countries have IPE activities that are integrated at several levels of their curricula. More thorough IPE curricula have been implemented at the University of the West of England, Bristol, United Kingdom [2], the University of British Columbia, Vancouver, Canada [8] and at Linköping University, Sweden [9-11]. These universities have introduced IPE curricula that span their entire educational programmes. In Australia and Canada, IPE activities are often included in educational activities located in rural areas [12,13]. By comparison, in the Nordic countries there are several examples where students participate in IPE activities on a hospital ward, often called an interprofessional training ward (IPTW), during the latter part of their training [14-20]. As a rule, IPE is supplied in a course/module for a few days or a couple of weeks during their training and participation by the students in these activities is often voluntary [5,21].

IPE is now spreading worldwide [22] and thus there is a need for studies focusing on the factors that affect students' interest in IPE activities. The aim of this study was to investigate whether student characteristics such as gender, previous working experience in healthcare, educational progress and such features of the learning environment as educational programmes and curriculum design have an impact on the students' readiness for interprofessional learning and how open-minded they are about co-operation with other professions.

## Methods

A cross-sectional study design was used. The Readiness for Interprofessional Learning Scale (RIPLS) was employed to measure students' readiness for interprofessional learning and their open-mindedness to co-operation with other professions [23,24]. Two universities in Sweden, both with undergraduate medical and nursing educational programmes, were chosen as sites for data collection.

### The Readiness for Interprofessional Learning Scale

**Figure 1:**
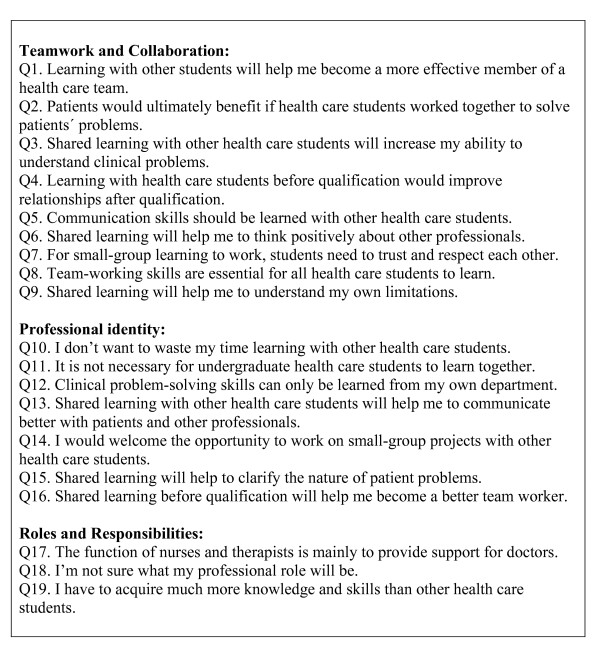
**The 19 items and the three factors on the Readiness for Interprofessional Learning Scale (RIPLS)**.

The RIPLS for evaluating interprofessional learning activities was originally presented by Parsell & Bligh [23,24]. The development of the RIPLS involved a conceptual framework based on evidence from the literature covering social and psychological theories and adult learning theory but also included professional expertise drawn from experiences in implementing interprofessional learning for healthcare students [23,24]. It consists of 19 items scored on a five-point Likert scale. All participants provide a score from 1 (completely disagree) to 5 (completely agree) for each of the 19 items. These items are then categorised into three main factors: Teamwork and Collaboration (Items 1-9), Professional Identity (Items 10-16) and Roles and Responsibilities (Items 17-19); see (Figure 1).

The scale has previously been used in different situations and for different student populations to evaluate interprofessional learning activities [1,25-29]. The Swedish version of the RIPLS, cross-culturally adapted to Swedish conditions and translated into Swedish [30], was used in this study.

The original factor structure was maintained when launching the Swedish version of the RIPLS [30]. However, in some studies where the RIPLS has been applied after its introduction, the factor structure and also some of the items have been suggested to be altered [29-31]. Initial tests showed that our data did not reflect the original factor structure of the RIPLS. Nor did the data support the internal consistency values of the subscales as previously reported. In the present study, we therefore decided to do a renewed factor analysis of the 19 items of the original RIPL Scale. Our factor analysis gave four item groupings instead of the originally proposed three and also a slightly different order of the 19 items. The factors were: Factor 1 (11 items, questions 1-6, 9 and 13-16), Factor 2 (4 items, questions 10-12 and 18), Factor 3 (2 items, questions 17 and 19) and Factor 4 (2 items, questions 7 and 8).

The internal consistency of the four factors was assessed by Cronbach's alpha as shown in Table 1. The internal consistency of the total RIPLS (all 19 items) gave a Cronbach's alpha of 0.62 (item mean, 3.57, and a min/max of 1.84/4.68). The Cronbach's alpha value of 0.88 for the cluster of items in factor 1 indicates a high internal consistency and that these 11 items represent a relatively unitary factor. An interprofessional expert panel of healthcare educators from the two participating universities scrutinised the semantic and conceptual essence of these 11 items. This resulted in an agreement to label this factor "Team Player". Thus a face validity of this concept was ensured. However, the re-ordering of the RIPL Scale suggested by the factor analysis of our empirical data was not satisfactory in terms of numbers of items in three of the factors (factors 2, 3 and 4), nor were satisfactory in terms of Cronbach's alpha values that were all below 0.60 for these three factors. The Cronbach's alpha value is generally recommended to be over 0.60 to consider a cluster of items as a genuine factor [32]. Therefore, we decided to omit these three clusters as factors, but the individual questions remain as single RIPLS items in the forthcoming analysis.

**Table 1:** Internal consistency of the four identified factors in this study of the Readiness for Interprofessional Learning Scale (RIPLS)

	Number of items	Cronbach's alpha	Item means	Min/max
**Factor 1**.	11	0.88	4.01	3.63/4.52

**Factor 2**.	4	0.51	2.14	1.98/2.31

**Factor 3**.	2	0.42	2.57	1.83/3.31

**Factor 4**.	2	0.38	4.66	4.65/4.67

The standardised maximum likelihood estimates of the factor loadings for the 11 items in the factor "Team Player" are displayed in (Table 2), as well as the items not included in this item group. The factor loading values for the items in the factor were acceptable.

**Table 2:** Standardised maximum likelihood estimates of the factor loadings for the 11 items included in the factor "Team Player" and items not included in this item group

**Factor 1. "Team player"**:	Factor loading
	

- Shared learning with other healthcare students will increase my ability to understand clinical problems (Q3).	0.71

- Shared learning will help me to think positively about other professionals (Q6).	0.71

- Shared learning with other healthcare students will help me to communicate better with patients and other professionals (Q13).	0.71

- Shared learning before qualification will help me become a better team worker (Q16)	0.70

- Shared learning will help to clarify the nature of patient problems (Q15).	0.70

- Learning with healthcare students before qualification would improve relationships after qualification (Q4).	0.69

- I would welcome the opportunity to work on small-group projects with other health- care students (Q14).	0.66

- Communication skills should be learned with other healthcare students (Q5).	0.65

- Shared learning will help me to understand my own limitations (Q9).	0.59

- Learning with other students will help me become a more effective member of a healthcare team (Q1).	0.58

- Patients would ultimately benefit if healthcare students worked together to solve patients' problems (Q2).	0.53

**Items not included in the "Team Player" item group**:

Q7. For small-group learning to work, students need to trust and respect each other.

Q8. Team-working skills are essential for all health care students to learn.

Q10. I don't want to waste my time learning with other health care students.

Q11. It is not necessary for undergraduate health care students to learn together.

Q12. Clinical problem-solving skills can only be learned from my own department.

Q17. The function of nurses and therapists is mainly to provide support for doctors.

Q18. I'm not sure what my professional role will be.

Q19. I have to acquire much more knowledge and skills than other health care students.

### Participants

For over 20 years, the "IPE University" has pursued an extensive interprofessional commitment offering an IPE curriculum to all their students in the health sciences. This university also utilises problem-based learning (PBL) as the pedagogical method [33,34]. All healthcare students are exposed to IPE activities for at least 12 weeks during their training, ranging from integrated courses in health, ethics and learning for 10 weeks both early on and in the middle of their educational programme to a final two weeks of interprofessional practice on a student training ward at the end of their professional training (in the 8^th ^semester for the medical students and the 6^th ^and last semester for the nursing students) [9-11,16,17]. The "IPTW University" has a mandatory two-week IPE course for nursing, medical, occupational therapy and physiotherapy students on interprofessional training wards. During this course the medical students are in their 8^th ^semester of 11 and the nursing students, as well all the other students, are in their 6^th ^and last semester [18,19]. The students are also offered other IPE activities (e.g. an IPE day in primary care, seminars in ethics) during their education, but these activities are voluntary and are usually available during the students' elective study periods [5].

At both universities students from medical and nursing programmes were invited to participate in the study. At the time of data collection, the medical students were starting their third or eighth semester and the nursing students their third or fifth or sixth semester. These semesters were deliberately chosen so that none of the students had participated in their upcoming two-week practice on the IPTWs. Students in semester 3 were labelled as "early" in their education in the analysis and students in semesters 5 to 8 were labelled as "late". The Research Ethics Committee of Linköping University, Sweden, approved the study (Dnr. 2010/26-31).

### Data Collection

The data collection was conducted in connection with introductory lectures at the universities for both the medical and nursing students at the beginning of the autumn semester in 2009. The students were informed both orally and in a written leaflet about the study and invited to fill in the questionnaire anonymously. After completion, the questionnaires were collected immediately. The questionnaire included a well-established psychometric instrument, RIPLS [23,24], and the following background variables: gender, age group, university, educational programme, stage of education and whether the student had any previous experience of working in healthcare.

### Statistical Methods

All data were stored in a database and analysed using the Statistical Package for the Social Sciences (SPSS) 18.0 software (Chicago, IL, USA). A factor analysis was applied to examine the factorial structure in our collected data from the previously translated Swedish version of RIPLS. Cronbach's alpha was used to assess reliability in terms of internal consistency. ANOVA was used thereafter for univariate tests between the independent variables and the items in the RIPLS, and mean and standard deviations were also calculated. Thereupon a multiple regression analysis was performed for each of the four identified factors, as well as for each of the 19 different questions in the RIPLS as dependent variables. The variables gender, medical vs. nursing programme, "IPE University" vs. "IPTW University", previous experience of working in healthcare, and stage of education were used as independent variables. A calculation of means and standard deviations for each of the four identified factors was done for all participants, without any weighting for individual items. Intercorrelation coefficients between the factors were calculated using Pearson regression analyses. A p value of < 0.05 was considered statistically significant.

## Results

A total of N = 955 students were invited to participate in the study, of which n = 670 filled in the questionnaire, giving an overall response rate of 70.2%. At the "IPE University" n = 378 students were invited and n = 299 responded (79.1%). At the "IPTW University" n = 577 students were invited and n = 371 participated (64.3%). Of the participating students, 56.6% (n = 379) came from the nursing programme and 43.4% (n = 291) from the medical programme. Among the participating students, 73.1% (n = 490) were females and 26.9% (n = 180) were males. The students at the "IPE University" were slightly younger (p = 0.026); 85.5% were under 30 years of age compared to 78.2% for the students at the "IPTW University". There were no statistically significant age differences between nursing and medical students or between female and male students. An overview of the participants in the study is presented in (Table 3).

**Table 3:** Study participants from the IPE University and the IPTW University

	IPE University n = 299		IPTW University n = 371		Totally N = 670
	**Early n**	**Late n**	**Early n**	**Late n**	**N**

**Medical programme**:					291

Females	40	23	57	38	158

Males	30	32	44	27	133

					

**Nursing programme**:					379

Females	89	64	74	105	332

Males	10	11	17	9	47

### Univariate Associations

Univariate correlations between the independent variables gender and educational programme and the factor "Team Player" and the other RIPLS items are shown in (Table 4). A high score (on the items Q1-Q6, Q9, Q13-Q16) indicates a positive attitude towards the "Team Player" concept. Females scored significantly higher on all "Team Player" items and also on other RIPLS items, except for question 18. Nursing students scored significantly higher on all "Team Player" items than the medical students. On the other hand, medical students scored significantly higher than nursing students on the single items Q10, Q11, Q17, Q18 and Q19. For the item Q7, nursing students scored significantly higher, while there was no difference for the items Q8 and Q12.

**Table 4:** Univariate associations between the variables gender and educational programme and the factor "Team Player" and the items in the RIPLS

	**Gender**:			**Educational programme**:		
	**Female (n = 490)**	**Male (n = 180)**		**Medical (n = 291)**	**Nursing (n = 379)**	

	**M (s.d.)**	**M (s.d.)**	**p**	**M (s.d.)**	**M (s.d.)**	**p**

**Factor index Team Player**:	4.16 (0.56)	3.81 (0.72)	0.0001	3.85 (0.66)	4.23 (0.55)	0.0001

Q 1	4.35 (0.79)	3.97 (1.02)	0.0001	4.08 (0.96)	4.39 (0.77)	0.0001

Q 2	4.59 (0.64)	4.34 (0.82)	0.0001	4.39 (0.76)	4.62 (0.64)	0.0001

Q 3	4.18 (0.86)	3.83 (0.97)	0.0001	3.79 (0.95)	4.31 (0.80)	0.0001

Q 4	4.52 (0.71)	4.29 (0.96)	0.001	4.39 (0.87)	4.52 (0.71)	0.033

Q 5	4.11 (0.86)	3.87 (0.96)	0.002	3.94 (0.95)	4.13 (0.84)	0.007

Q 6	4.12 (0.93)	3.58 (1.11)	0.0001	3.64 (1.12)	4.24 (0.82)	0.0001

Q 9	3.86 (0.88)	3.56 (0.96)	0.0001	3.66 (0.94)	3.87 (0.88)	0.004

Q 13	4.17 (0.85)	3.84 (1.04)	0.0001	3.92 (0.92)	4.21 (0.90)	0.0001

Q 14	3.78 (1.16)	3.27 (1.30)	0.0001	3.20 (1.30)	3.97 (1.05)	0.0001

Q 15	3.82 (1.01)	3.47 (1.08)	0.0001	3.37 (1.06)	4.01 (0.94)	0.0001

Q 16	4.25 (0.86)	3.85 (1.02)	0.0001	3.94 (0.97)	4.29 (0.84)	0.0001

						

**RIPLS Items**:						

Q 7	4.73 (0.53)	4.49 (0.80)	0.0001	4.60 (0.67)	4.72 (0.59)	0.012

Q 8	4.71 (0.54)	4.49 (0.74)	0.0001	4.65 (0.58)	4.65 (0.63)	0.960

Q 10	1.93 (1.11)	2.22 (1.17)	0.004	2.21 (1.09)	1.85 (1.14)	0.0001

Q 11	2.23 (1.12)	2.60 (1.13)	0.0001	2.56 (1.12)	2.14 (1.12)	0.0001

Q 12	1.93 (1.11)	2.13 (1.10)	0.036	2.05 (1.03)	1.93 (1.17)	0.165

Q 17	1.71 (1.02)	2.13 (1.18)	0.0001	2.06 (1.11)	1.65 (1.03)	0.0001

Q 18	2.25 (1.16)	2.34 (1.12)	0.360	2.46 (1.15)	2.14 (1.13)	0.0001

Q 19	3.06 (1.33)	3.99 (1.19)	0.0001	4.31 (0.87)	2.56 (1.16)	0.0001

						

Univariate correlations between the independent variable type of university and the RIPLS items are shown in Table 5. There were no significant differences between the two universities regarding the factor "Team Player". However, for some of the single items in the "Team Player" factor (Q1, Q2 and Q9), students from the "IPE University" scored significantly higher, while students from the "IPTW University" scored higher on the single item Q14. For the rest of the RIPLS items, the "IPE University" students scored higher on item Q7, while the students from the "IPTW University" scored significantly higher on item Q11. Students in the early stage of their education scored significantly higher on Q13, Q14, Q17, Q18 and Q19 on the RIPLS than students in the later stage of their education.

**Table 5:** Univariate associations between the variables gender and educational programme and the factor "Team Player" and the items in the RIPLS

	**University**:			**Early or late in education**:		
	**IPE University (n = 299)**	**IPTW University (n = 371)**		**Early (n = 361)**	**Late (n = 309)**	

	**M (s.d.)**	**M (s.d.)**	**p**	**M (s.d.)**	**M (s.d.)**	**p**

						

**Factor index "Team Player"**:	4.07 (0.59)	4.07 (0.66)	0.990	4.10 (0.59)	4.03 (0.66)	0.155

Q 1	4.33 (0.81)	4.19 (0.91)	0.032	4.22 (0.85)	4.28 (0.89)	0.375

Q 2	4.63 (0.59)	4.43 (0.77)	0.0001	4.53 (0.70)	4.51 (0.70)	0.664

Q 3	4.15 (0.87)	4.04 (0.93)	0.111	4.09 (0.88)	4.09 (0.93)	0.949

Q 4	4.46 (0.72)	4.47 (0.83)	0.825	4.47 (0.76)	4.46 (0.82)	0.852

Q 5	3.99 (0.86)	4.10 (0.92)	0.112	4.10 (0.86)	4.00 (0.93)	0.147

Q 6	3.93 (1.02)	4.01 (0.99)	0.323	3.99 (0.94)	3.95 (1.08)	0.611

Q 9	3.86 (0.84)	3.71 (0.96)	0.031	3.82 (0.88)	3.73 (0.95)	0.179

Q 13	4.05 (0.93)	4.11 (0.91)	0.399	4.16 (0.84)	4.00 (0.99)	0.019

Q 14	3.45 (1.21)	3.80 (1.22)	0.0001	3.77 (1.11)	3.50 (1.33)	0.004

Q 15	3.72 (0.99)	3.74 (1.08)	0.756	3.76 (0.98)	3.69 (1.11)	0.375

Q 16	4.15 (0.92)	4.14 (0.93)	0.891	4.17 (0.88)	4.11 (0.97)	0.452

						

**RIPLS Items**:						

Q 7	4.75 (0.49)	4.60 (0.71)	0.001	4.69 (0.64)	4.64 (0.61)	0.356

Q 8	4.69 (0.56)	4.62 (0.64)	0.137	4.63 (0.63)	4.67 (0.59)	0.319

Q 10	1.95 (1.13)	2.04 (1.14)	0.285	2.06 (1.14)	1.93 (1.13)	0.158

Q 11	2.21 (1.16)	2.42 (1.11)	0.018	2.38 (1.12)	2.26 (1.15)	0.186

Q 12	1.95 (1.03)	2.01 (1.17)	0.480	2.01 (1.07)	1.95 (1.16)	0.487

Q 17	1.87 (1.09)	1.79 (1.07)	0.348	1.91 (1.08)	1.72 (1.07)	0.021

Q 18	2.22 (1.11)	2.33 (1.19)	0.214	2.42 (1.16)	2.10 (1.12)	0.0001

Q 19	3.27 (1.37)	3.34 (1.35)	0.547	3.42 (1.30)	3.17 (1.41)	0.017

There were no statistically significant differences (data not shown) in the RIPLS items between students with previous experience of work in the healthcare sector and students without such experience.

#### Multivariate Associations

The multiple regression analysis revealed independent and statistically significant associations between the factor "Team Player" and females, students in the nursing programme and students in early training; see (Table 6). The variables gender and educational programme showed an independent and significant association for most of the 11 items in this factor. The variables early vs. late stage in education and type of university showed an independent and significant association for a few items in the "Team Player" factor.

**Table 6:** Multiple regression analysis of different independent variables associations with the factor "Team Player" (11 items from the RIPLS scale)

	Gender: female vs. male	Educational programme: medical vs. nursing	IPE University vs. IPTW University	Early vs. late in education	Previous experience of healthcare work
	**Beta p value**	**Beta p value**	**Beta p value**	**Beta p value**	**Beta p value**

					

**Factor index "Team Player"**:	(-0.201) 0.001	(0.291) <0.0001	(0.013) 0.786	(-0.110) 0.026	(-0.083) 0.091

Item Q 1	(-0.272) 0.001	(0.179) 0.017	(-0.122) 0.081	(0.044) 0.533	(-0.078) 0.262

Item Q 2	(-0.151) 0.026	(0.158) 0.009	(-0.215) <0.0001	(-0.040) 0.480	(-0.042) 0.447

Item Q 3	(-0.143) 0.100	(0.479) <0.0001	(-0095) 0.187	(-0.045) 0.532	(-0.105) 0.144

Item Q 4	(-0.187) 0.017	(0.039) 0.572	(0.040) 0.532	(-0.045) 0.485	(-0.086) 0.182

Item Q 5	(-0.160) 0.071	(0.093) 0.240	(0.137) 0.062	(-0.165) 0.025	(-0.107) 0.146

Item Q 6	(-0.300) 0.002	(0.486) <0.0001	(0.110) 0.164	(-0.185) 0.019	(-0.010) 0.898

Item Q 9	(-0.245) 0.007	(0.107) 0.180	(-0.159) 0.033	(-0.052) 0.481	(0.080) 0.280

Item Q 13	(-0.234) 0.011	(0.200) 0.014	(0.100) 0.185	(-0.225) 0.003	(-0.060) 0.429

Item Q 14	(-0.144) 0.209	(0.746) <0.0001	(0.344) <0.0001	(-0.334) <0.0001	(-0.154) 0.105

Item Q 15	(-0.098) 0.323	(0.588) <0.0001	(0.069) 0.406	(-0.126) 0.126	(-0.214) 0.009

Item Q 16	(-0.258) 0.004	(0.185) 0.021	(-0.028) 0.712	(-0.060) 0.423	(-0.087) 0.245

(All multiple regression models, df = 5 and p < 0.0001 except model for item Q4 (p = 0.051, F = 2.22) and item Q5 (p = 0.006, F = 3.26) and item Q9 (p = 0.002, F = 3.84)

## Discussion

We set out to investigate whether student characteristics such as gender, previous working experience in healthcare, educational progress and such features of the learning environment as educational programmes and curriculum design have an impact on the students' readiness for interprofessional learning and how open-minded they are about co-operation with other professions. The main findings were that female students in general and nursing students had a more positive attitude to teamwork. did Exposure to different interprofessional curricula was only to a minor extent associated with the students' attitudes to teamwork, and educational progress did not seem to alter these beliefs.

The finding that female students appear to take a more positive view of teamwork has also been reported in another recent Swedish study [3]. In many respects, young Swedish women of today grow up in a more democratic and egalitarian society, a society that has strengthened women's position. They convey an image of being equal to males and have expectations of working in such a way also in their occupation. However, the Swedish healthcare system still maintains traditional hierarchical structures, even though it is transitioning towards more teamwork and more patient-centred care. Women appear to be more willing to change the hierarchical healthcare system - a system built by men for men [6]. This might be due to the fact that hierarchical organisations often give women fewer opportunities to influence their working conditions.

Actually, women constitute the majority of medical students in Sweden today. In nursing education, the numbers of men are steadily increasing, even though they are still in the minority, i.e. only about 10%. There are also differences in perspectives between these two educational programmes: nursing education in Sweden covers both behavioural (50%) and biological (50%) sciences, while 90% of the medical education is biologically oriented [35]. The differences in perspectives between the programs may explain that nursing students seemed to welcome teamwork and collaboration more than medical students. Such an interpretation is also in accordance with previous reports [1].

In other previous Swedish studies, medical students have reported scepticism about IPTWs after a two-week placement, expressing the view that the aim of the training was in conflict with their ambition to take on their new roles as physicians [14,19]. Medical students' perceptions such as "I do not want to waste my time attending courses together with others" (Item 10) and "Other professions in healthcare have support functions to the doctor" (Item 17), as expressed in this study, are counter-productive to teamwork. The students at the "IPE University", who had been exposed to interprofessional education [10,11] before the survey was completed, reported more positive attitudes towards teamwork in 4 out of 11 items in the factor "Team Player", compared to the students at the "IPTW University". However, it should be noted that the students at the "IPE University" also had been exposed to a problem-based curriculum [25,26] and therefore were well acquainted with working in small groups.

It is possible that there are other important factors that influence the attitudes and beliefs about co-operation which we did not cover using the RIPLS. Other factors that might be crucial and important for willingness to co-operate and participate in teamwork could involve the personality of the individual. Forthcoming studies will include measurements of personality, which might be a way to enhance our understanding of interprofessional learning and competence.

### Methodological Considerations and Limitations of the Study

In a previous study in which the British RIPLS was tested for Swedish conditions, it was concluded that further analysis with other empirical material could enhance the factor structure and improve the model [30]. In the initial factor analysis of our collected data, four groupings of items were identified instead of three in the original scale. Our renewed factor analysis also indicated another order of the items in each factor. Our student sample was three times larger (n = 670 participants) than in the previous Swedish study [30] with only 214 participants, which may have had an impact on the factor analysis. General statistical recommendations concerning factor analysis often suggest that item groupings with a Cronbach's alpha under 0.60 should not constitute a factor [32]. In our study the Cronbach's alpha for three of the groupings was less than 0.60. Therefore, we decided to omit these three as factors and treat the RIPLS items included in these groupings as single questions in the analysis. In agreement with other researchers [29-31], we believe that there is room for improvements and adjustments in the RIPLS. Our main contribution to this issue is the introduction of a factor that we labelled "Team Player". The denotation of this concept was decided upon after a face validity analysis performed by an interprofessional expert panel of healthcare educators, taking both the semantics and the conceptual essence of the factor into consideration. Nonetheless, "Team Player" should still be regarded as a tentative concept and its validity needs to be finally established in forthcoming research. Theoretically, a "Team Player" in healthcare acts co-operatively with other professionals, has a complementary background, skills in the dynamic "teamwork" process, and shares common goals [36]. However, illustrative examples of the concept are above all found in sports, where a team player is known to sacrifice personal achievements in order to help the team win.

Possible limitations of the study include the participation rates, a risk of mass significance, and an uneven gender distribution among the respondents. Although the overall response rate was over 70%, which is quite acceptable, the participation rates for the students at the "IPTW University" were lower (64%), which might have influenced the results. The fact that there are 19 single items in the RIPLS and several independent variables means that a number of significant tests were performed. This might raise the risk of mass significance. Therefore, significant differences for individual items on the scale should be interpreted with some caution, especially if the significance levels were close to the borderline (p = 0.05) for an accepted level. The uneven distribution of gender among the participants in this study, with two thirds being female and only one third males could influence the generalisability of the study. However, this phenomenon reflects the actual male/female ratios today in Sweden for the two studied educational programmes. Students in the nursing programme are predominantly females, but today the gender distribution in the medical programme is more even, with a slight predominance of females.

## Conclusions

The main findings in this study were that female students in general and nursing students had a more positive view of interprofessional learning and were more open-minded about co-operation with other professions. Exposure to a more extensive interprofessional curriculum was only to a minor extent associated with a positive attitude towards teamwork. Nor did educational progress change these beliefs.

This study indicates some directions for more successful interprofessional education. Efforts should be directed at informing particularly male medical students about the need for teamwork in modern healthcare systems. The results also imply that study of other factors, such as the student's personality, is needed to fully understand readiness for teamwork and interprofessional collaboration in healthcare. We also believe that the RIPL Scale still can be further adjusted.

## Competing interests

The authors declare that they have no competing interests.

## Authors' contributions

MW, TF and SP participated in the study design. MW co-ordinated and completed the data collection and drafted the first manuscript. All authors contributed to the analysis and interpretation of the data and writing of the manuscript and approved the final version.

## Pre-publication history

The pre-publication history for this paper can be accessed here:

http://www.biomedcentral.com/1472-6920/11/15/prepub

## References

[B1] Hind M Norman I Cooper S Gill E Hilton R Judd P Jones SC Interprofessional perceptions of health care students Journal of Interprofessional Care 2003 17 1 21 34 10.1080/1356182021000044120 12772467

[B2] Pollard KC Miers M From students to professionals: Results of a longitudinal study of attitudes to pre-qualifying collaborative learning and working in health and social care in the United Kingdom Journal of Interprofessional Care 2008 22 4 399 416 10.1080/13561820802190483 18800281

[B3] Hansson A Foldevi M Mattsson B Medical students' attitudes toward collaboration between doctors and nurses - a comparison between two Swedish universities Journal of Interprofessional Care 2010 24 3 242 250 10.3109/13561820903163439 19995272

[B4] Mandy A Milton C Mandy P Professional stereotyping and interprofessional education Learning in Health and Social Care 2004 3 154 170 10.1111/j.1473-6861.2004.00072.x

[B5] Lewitt MS Ehrenborg E Scheja M Brauner A Stereotyping at the undergraduate level revealed during interprofessional learning between future doctors and biomedical scientists Journal of Interprofessional Care 2010 24 1 53 62 10.3109/13561820902921704 20001546

[B6] Zelek B Philips SP Gender and power: Nurses and doctors in Canada International Journal for Equity in Health 2003 2 1 10.1186/1475-9276-2-1 12605720 PMC150379

[B7] Ivanova-Stenzel R Kübler D Courtesy and Idleness: Gender Differences in Team Work and Team Competition SFB 649, Homboldt-University in Berlin 2005 http://sfb649.wiwi.hu-berlin.de ISSN 1860-5664

[B8] Grant C Brainbridge L Gilbert J The University of British Columbia model of interprofessional education Journal of Interprofessional Care 2010 24 1 9 18 10.3109/13561820903294549 20001544

[B9] Areskog NH The new medical education at the Faculty of Health Sciences, Linköping University - a challenge for both students and teachers Scandinavian Journal of Social Medicine 1992 20 1 1 4 1585134 10.1177/140349489202000101

[B10] Areskog NH Undergraduate interprofessional education at Linköping Faculty of Health Sciences - How it all started Journal of Interprofessional Care 2009 23 5 448 454 10.1080/13561820903163579 20602584

[B11] Wilhelmsson M Pelling S Ludvigsson J Hammar M Dahlgren LO Faresjö T Twenty years experiences of interprofessional education in Linköping - ground-breaking and sustainable Journal of Interprofessional Care 2009 23 2 121 133 10.1080/13561820902728984 19225972

[B12] Lee A Dunston R Nisbe G Matthews L Pockett R Interprofessional developments in Australia - L-TIPP (Aus) and the Way Forward Journal of Interprofessional Care 2009 23 3 315 317 19517281 10.1080/13561820903028251

[B13] Faresjö T Interprofessional education - to break boundaries and build bridges Rural Remote Health 2006 6 3 602 16827613

[B14] Fallsberg MB Wijma K Student's attitudes towards the goals of an interprofessional training ward Medical Teacher 1999 21 6 576 581 10.1080/01421599978997 21281177

[B15] Fallsberg MB Hammar M Strategies and focus at an integrated, interprofessional training ward Journal of Interprofessional Care 2000 4 339 350

[B16] Wahlström O Sanden I Hammar M The student ward at the University Hospital, Faculty of Health and Sciences, Linköping European Nurse 1996 1 262 267

[B17] Wahlström O Sandén I Multiprofessional training ward at Linköping University, early experience Education for Health 1998 11 225 231

[B18] Hylin U Nyholm H Mattiasson AC Ponzer S Interprofessional training in clinical practice on a training ward for healthcare students: a two-year follow-up Journal of Interprofessional Care 2007 21 3 277 288 10.1080/13561820601095800 17487706

[B19] Ponzer S Hylin U Kusoffsky A Lauffs M Lonka K Mattiasson AC Nordström G Interprofessional training in the context of clinical practice: goals and student's perceptions on clinical education wards Medical Education 2004 38 7 727 736 10.1111/j.1365-2929.2004.01848.x 15200397

[B20] Jacobsen F Fink AM Marcussen V Larsen K Hansen BæK T Interprofessional undergraduate clinical learning: Results from a three year project in a Danish Interprofessional Training Unit Journal of Interprofessional Care 2009 23 1 30 40 10.1080/13561820802490909 19142781

[B21] Anderson E Lennox A The Leicester Model of Interprofessional Education: Developing, delivering and learning from student voices for 10 years Journal of Interprofessional Care 2009 23 6 557 573 10.3109/13561820903051451 19842950

[B22] World Health Organization Framework for action on Interprofessional Education and Collaborative Practice 2010 WHO; Geneva 21174039

[B23] Parsell G Bligh J The development of a questionnaire to assess the readiness of health care students for interprofessional learning (RIPLS) Medical Education 1999 33 95 100 10.1046/j.1365-2923.1999.00298.x 10211258

[B24] Parsell G Bligh J Educational principles underpinning successful shared learning Medical Teacher 1998 20 6 522 529 10.1080/01421599880229

[B25] Horsburgh M Lamdin R Williamson E Multiprofessional learning: the attitudes of medical, nursing and pharmacy students to shared learning Medical Education 2001 35 9 876 883 10.1046/j.1365-2923.2001.00959.x 11555226

[B26] Mackay S The role perception questionnaire (RPQ): a tool for assessing undergraduate students' perceptions of the role of other professions Journal of Interprofessional Care 2004 18 3 289 302 10.1080/13561820410001731331 15369972

[B27] Baxter S Perspectives and practice: speech and language therapy student views of an interprofessional learning experience Learning in Health and Social Care 2004 3 2 102 110 10.1111/j.1473-6861.2004.00065.x

[B28] Morison S Boohan M Moutray M Jenkins J Developing pre-qualification interprofessional education for nursing and medical students: sampling student attitudes to guide development Nurse Education in Practice 2004 4 1 20 29 10.1016/S1471-5953(03)00015-5 19038133

[B29] Reid R Bruce D Allstaff K McLernon D Validating the Readiness for Interprofessional Learning Scale (RIPLS) in the postgraduate context: are health care professionals ready for IPL? Medical Education 2006 40 415 422 10.1111/j.1365-2929.2006.02442.x 16635120

[B30] Lauffs M Pontzer S Saboonchi F Lonka K Hylin U Mattiasson AC Cross-cultural adaption of the Swedish version of Readiness for Interprofessional Learning Scale (RIPLS) Medical Education 2008 42 405 411 10.1111/j.1365-2923.2008.03017.x 18338993

[B31] McFadyen AK Webster V Strachan K Figgins E Brown H McKechnie J The Readiness for Interprofessional Learning Scale: A possible more stable sub-scale model for the original version of RIPLS Journal of Interprofessional Care 2005 19 6 595 603 10.1080/13561820500430157 16373215

[B32] Shea S Fortna G Norman GR, van der Vleuten CPM, Newble DI Psychometric methods International Handbook of Research in Medical Education, Part 1 2002 London, Kluwer Academic Publishers 97 126

[B33] Silén S Assisting Learning - the tutor role in Problem Based Learning 1996 3 Linköping: Faculty of Philosophy, Linköping University Thesis nr

[B34] Silén S Uhlin L Self-directed learning - an issue for students and the faculty! Teaching in Higher Education 2008 13 4 461 475 10.1080/13562510802169756

[B35] Hultberg J Rosenberg C Thorpenberg S Nordholm L Elzinga A Brogren PO Samuelsson B A Model of the Study of Research and Education and Transdisciplinary Context Knowledge, Technology and Policy 1998 11 1&2 167 190

[B36] Xyrichis A Ream E Teamwork: a concept analysis Journal of Advanced Nursing 2008 62 232 241 10.1111/j.1365-2648.2007.04496.x 18186914

